# Effect of the Affordances of the FM New Media Communication Interface Design for Smartphones

**DOI:** 10.3390/s21020384

**Published:** 2021-01-08

**Authors:** Hongyu Li, Chien-Hsiung Chen

**Affiliations:** Department of Design, National Taiwan University of Science and Technology, Taipei 10607, Taiwan; cchen@mail.ntust.edu.tw

**Keywords:** new media, perception, FM, affordance, smartphone, interface design

## Abstract

Smartphone equipment has promoted the widespread use of new media communication, and users have changed from being passive to actively receiving information. This has changed people’s lifestyles, and has enriched the convenience and entertainment value of knowledge acquisition. However, the new media communication systems can be too complex, and the interface design for users to interact with the new media may not be sufficiently intuitive, which causes interface usability problems. Therefore, this research study focuses on the concept of affordance and the impact of user perception pertinent to smartphone applications on frequency modulation (FM) new media interface design. The experiment is a between-subjects design using one-way ANOVA to examine three different operation modes, namely the Litchi, Himalayas and Archimedes types. The experimental data were obtained through task performance and subjective evaluation. The results indicate that: (1) Visual information presentation methods, such as viewing and deleting, affect user perception. The three operating modes revealed significant differences, with the Himalayas type taking the least amount of task performance time. (2) There were significant differences among the different operation modes, with the Himalayas type the best in terms of the users’ subjective evaluation. (3) The overall analysis of task performance and satisfaction consistently showed that the Himalayas type was better than the Litchi and Archimedes types in all aspects. (4) Smartphone user interface applications provide users with cognitive support, objects, functions, and sensory affordance, which enhance the user’s interactive experience of FM new media.

## 1. Introduction

With the birth and development of mobile media and new types of smart equipment, especially smartphone mobile equipment, the quality of new media communication has increased. This has changed people’s lifestyles, and the users’ original media contact time and habits have been disrupted, while the fragmentation and dissemination of information have also cultivated new reading and audiovisual habits [1]. On the one hand, the fragmentation of listening time and content enables users to listen across time and space. However, the traditional listening mode requires a fixed time to hear the relevant broadcast information. With traditional radios, it is necessary to play and hear the information at a fixed time, the positioning of the program is uncertain, the information content tends to be singular, and the range of choices is narrow. The listener passively accepts a large amount of input information and cannot listen to the information according to his/her personal preferences. In contrast, the smartphone frequency modulation (FM) new media listening platform has a large capacity through the “cloud” processor, and the information content is rich and high-quality, including audiobooks, language learning, and emotional columns, with information sourced from home and abroad. Its customization, personalization, and diversification have become a trend [2]. The FM new media uses a smartphone application (app) to operate tasks that are simple, intuitive, and easy to perform. It can provide the latest news anytime and anywhere according to users’ personal preferences. In terms of listening content, each app has its own unique features. Some products are positioned as “sound” interaction and the content division is more focused, such as Litchi (i.e., type, style, scene, age) and Archimedes. They have attracted many professional audio enthusiasts and cultivated a large number of excellent anchors, but at the same time, it has also led to limited audiences. Some products are more diversified in terms of positioning. In addition to voice interaction, they also include live broadcast and audio. For example, the information included in Himalaya FM is more systematic and comprehensive. In the learning category alone, there are speeches, foreign language learning, cross talk, celebrity information, and so on, and paid channels have also been opened. These have not only expanded the audience’s attention but have also heavily invested in continuously attracting users to build a high-quality listening platform. Traditional broadcasting mainly involves linear transmission. Due to the limitations of time and space, it is impossible to listen to one’s personal favorite information in a very short amount of time. Spreaders play a leading role due to their advantages in terms of resources and technology. Although the audience can participate in the interaction by calling in, there are few user interactions available, and users cannot participate simultaneously. Sometimes, the interaction is disrupted by busy lines. The FM new media can enhance the interactivity with the help of digital technology, and update the audience with timely feedback. This can help break down the traditional antagonism between transmission and reception [3]. The development of digital technology has given birth to the vigorous development of new media functions, and produced various new media types. It has also provided convenience for the majority of users. The combination of old media and new technology makes conveying intentions more convenient.

Given the transformation of media, audience identity has become a highlight of new media product competition. Users have actively switched from traditional passive participation to listening to information across time and space anytime and anywhere. At the same time, new media have also contributed to the improvement of user subjectivity, emphasizing user experience as the current social consensus of the Internet [4]. Interface design serves as an intermediate portal between users and new media products, which means that users establish an intuitive interaction bridge through a smartphone app and FM new media platform. Users can visually perceive product content through the user interface. It shows that the affordance of the transformation from the traditional to the virtual space-time environment affects the success or failure of new FM media products. This conversion also generates a number of difficulties for users. First, users break away from traditional interaction styles and transform into active participants when thinking about searching for information, which adds an invisible burden for users. Second, as the current development of new media products may still be in its early stage, there are many inconveniences in task operation, the content can be complicated, and the recognition of functional icon elements can be weak. A good user interface can help users avoid these problems and guide them to understand the smooth interaction of products [5]. Affordance design in the virtual environment interaction process can strengthen the users’ subjective initiative, and highlight the meaning of product functions [6]. Norman [7] indicated in the principle of interaction design that affordance is the core foundation of product design. Klügl [8] believes that affordance forms the foundational idea that the user must perceive what he/she can do with a particular element. Zhao et al. [9] perceived affordance of diversity in which new media interaction design should reflect its characteristics, and emphasized that it can promote the basic element design of new media.

### 1.1. Affordance Concept

**Figure 1:**
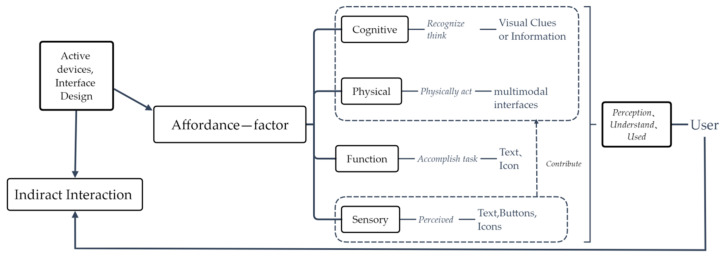
Conceptual diagram of interaction design pertaining to the affordance factor.

The term affordance originated from Gibson’s perceptual psychology which proposed that animals perceive the environment to provide behavioral possibilities, emphasizing the behavioral relationship between them, and reflecting the subjective/objective dichotomy [10]. With the development of modern social patterns and technology, the term affordance has become more abundant and widely used in its conceptual meaning. Evans et al. [11] systematically combined and analyzed from the perspective of media communication to clarify the development of the concept of affordance. They suggested that it can be defined according to the research orientation, emphasizing the consistency of the cited conception and application, and expanding its operability definition. They also pointed out that the development of the concept of affordance should follow threshold standards, namely Anonymity, Persistence, and Visibility. Their research results also show that the creation of affordance has positive significance for the application of new media communication. Norman [7] proposed perceptual affordance based on changes in people’s living environment, which helps highlight the hidden meaning of artifacts and enhances intuitive interaction. It is a broad application in the HCI field, and its concept and application are consistent. Gaver [12,13] put forward the term technological affordance, emphasizing the grouping support of time and space, which helps generate action through technical interaction. Hartson [14] further expanded the concept of affordance and identified four types, namely cognition, object, function, and sensory affordances. Based on the analysis of video media, Van Osch and Mendelson [15] proposed the types of affordance among developers, users, and artifacts. Besides, the cross integration of new media and interactive design language will further broaden the development of this concept. New media serve as a form of social interaction and provide space for communication [9]. It has also been shown that new media products not only need to strengthen the usability of design but also need to inject the sociality of emotional needs to improve user cognition. Given the consistency of concept and application, this study is based on the concept of perceptual affordance proposed by Norman [7]. Through the expansion of the conception of affordance and its four types in Hartson [14], this study aimed to investigate the impact of affordance design on FM new media and the effect of interface design. Figure 1 shows the conceptual diagram of interaction design pertaining to the affordance factor generated in this study.

### 1.2. Affordances in a New Medium

With the change in communication methods pertaining to new media, users have changed from performing passive to active activities. Users tend to enter and receive commands through smart devices, and interact with their virtual interfaces. The affordance provided by active devices can directly affect the usability and satisfaction of smart media [16]. The smart interfaces of new media have a variety of interface forms to establish interaction with users, such as touch graphics, voice control, and gestures. Therefore, the user interface of new media should provide reasonable capabilities in terms of vision, hearing, and touch to improve its usability. Users implement tasks through the interface icons on the touch screen. The graphics are the main elements for the human-computer interaction. Only well-designed and reasonable icons can convey the intent of the complex system [17]. It is conducive to understanding, learning, and attracting attention and other advantages [18,19,20] to help achieve a multiplier effect. Hartson [14] expanded the four types of affordance, namely function, object, cognition, and perception, which can effectively promote the interaction of new media interfaces, help users operate tasks more conveniently, obtain the required services, and play a positive guiding role. The design features of cognitive affordance (i.e., visual cues or information) can help users understand and recognize how a task is performed. The lack of affordance clues can lead to reduced search and click-to-action buttons on the display. However, the depth and contrast on the buttons (or both) improve search performance [21]. Object affordance helps users take action more easily when carrying out tasks. Functional affordance helps user complete tasks. Sensory affordance helps users perceive the characteristics of objects. The application of these four types of affordance in the new media interface design is mutually integrated, which can improve the usability of direct interaction between smart devices and users. Norman [7] indicated that interaction design should follow the six principles of design to help user complete tasks quickly; these principles are Visibility, Feedback, Constraints, Mapping, Consistency, and Affordance. He also emphasized that the content of the interface design should be clear and concise to help reduce unnecessary information, and that it should fully mobilize the invisible implications of the graphics or icons, which is beneficial to providing a sense of affordance. This would also help users easily use the new media interface, reduce their psychological load, and reduce their perceived uncertainty.

### 1.3. Interaction Design of FM New Media

The interface of smart devices provides a variety of interaction possibilities for new media communication and highlights usability. However, usability focuses on the intuitiveness and ease of learning in the interaction between people and products [22]. As a platform to support and promote social activities, communication, and collaboration, new media communication must consider usability and guide users to participate in the social interaction. Usability attracts designers’ attention, while sociality brings together multiple identities to participate [23]. Zhao et al. [9] established three crucial components of social media design, namely social media as an IT artifact, user type, and the role in social media application domains. They respectively refer to the emphasis on content and form, the emphasize user types in social media interaction design and the solving of specific problems or presenting different functions. They believe that the concept development is multi-dimensional, and social benefits are brought by the comprehensive framework. These are all conducive to our comprehensive understanding of the application of affordance design on the new media virtual platform.

## 2. Research Objective and Questions

The purpose of this study is to investigate the user interface design of FM new media pertinent to smart operation mode. The experiment was designed to investigate how “operation mode” (i.e., the Litchi, Himalayas, and Archimedes types) on the Smart FM new media interface would affect users’ task performance, system usability (measured using the system usability scale [SUS]) and their subjective evaluations.

We raised the following four questions:

- Q1: Do the different operation modes of user interface designs significantly affect user task performance and their subjective evaluations?
- Q2: Do visual cues affect participants’ perception of affordance?
- Q3: How does the application of the four types of affordance affect the design of new media interfaces?
- Q4: Do participants who spend the shortest time tend to be most satisfied with the Himalayas type of interface design?

## 3. Method and Materials

**Figure 2:**
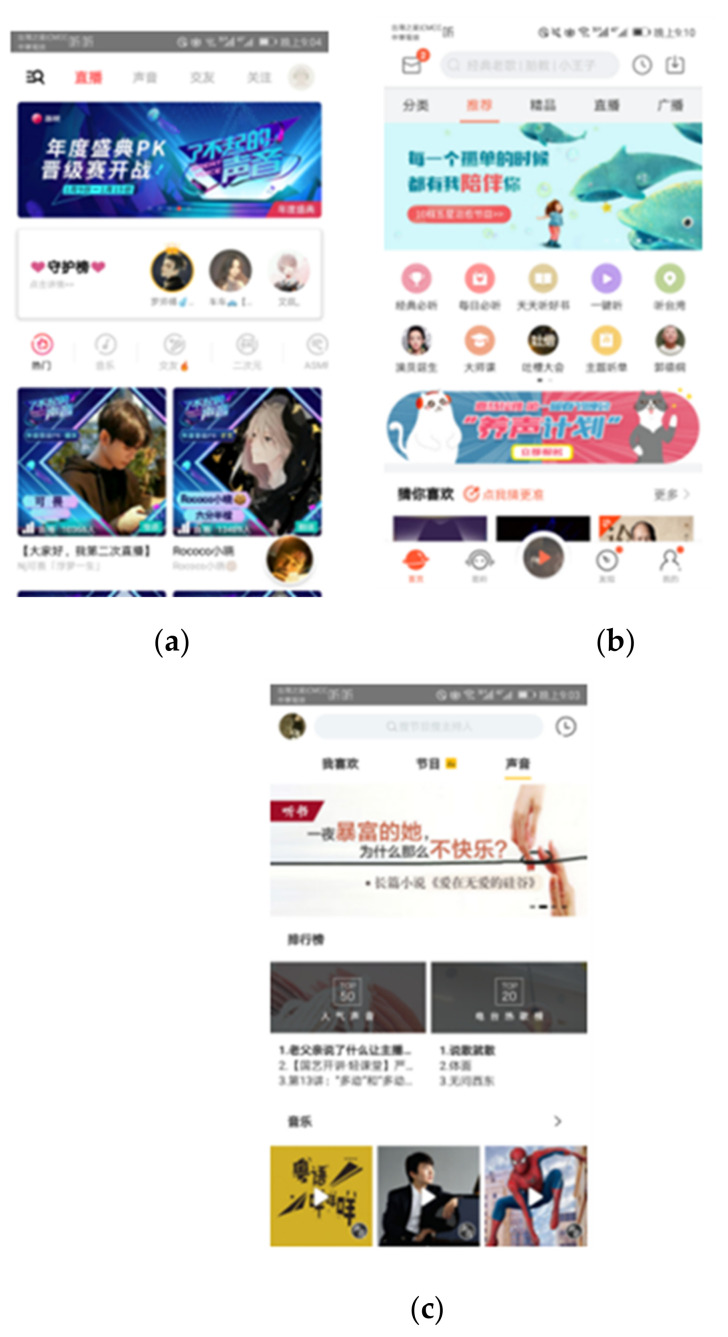
Three user interface designs of the FM touch screens: (**a**) Litchi type; (**b**) Himalaya type; (**c**) Archimedes type.

The experiment adopted a one-way ANOVA design. The independent variable is “operation mode”. It is a between-subjects factor, and the three levels were the Litchi, Himalayas and Archimedes types. The dependent variables were the users’ task performance and scores from SUS and subjective evaluations. The study tasks included five items for the purpose of investigating the participants’ behavior in the new media operating environment. We implemented them on a HUAWEI P10 smartphone (HUAWEI, Shenzhen, China) with a 5-inch screen. According to online research, from traffic, rankings, downloads, and audiences, these three apps rank high on the FM platform, especially Himalaya and Lychee, which have certain influence and popularity. The content of the three types is distinctive and representative. They have similar functions for the experimental tasks. That is, the three user interfaces are all smartphone apps, among which the Litchi type is a professional FM platform that focuses on “sound” and its interface content modules are more related to arts and entertainment information. The Himalayas type is a FM new media platform with more systematic and comprehensive functionality and content integration. The interface information is rich and diverse, and has a great deal of social influence. The Archimedes type is a new media platform which falls between the professional and mass FM new media with many stations. All the three apps were very popular in the market based on top on-line rankings and download rates (see Figure 2).

The experimental results were analyzed based on the one-way ANOVA. That is, by using the SPSS software (IBM, Armonk, NY, USA), the participants’ task completion time, SUS scores, and subjective evaluations were analyzed. Significant main effects were further analyzed by post hoc comparisons.

### 3.1. Participants

The convenience sampling method was used in this study because common mobile device users may use the FM new media and they are all potential participants. Therefore, there is no specific restriction in terms of participants. A total of 30 users participated in this experiment (i.e., nine males, 21 females). Their education level was above bachelor’s degree, and their age was between 20 and 40 years old. They all had experience in using mobile apps. Nonetheless, their experience level was assessed based on their background information. Among them, 14 participants did not use the FM new media before, seven of them with one year of use experience, and nine of them with more than two years of use experience. Based on the above-mentioned information, 46.7% (i.e., 14 participants) of them were viewed as having lower use experience, while 53.3% (i.e., 16 participants) of them as having higher use experience. According to the distribution of data, most people had used the app and could complete the experiment independently.

### 3.2. Procedure

**Figure 3:**
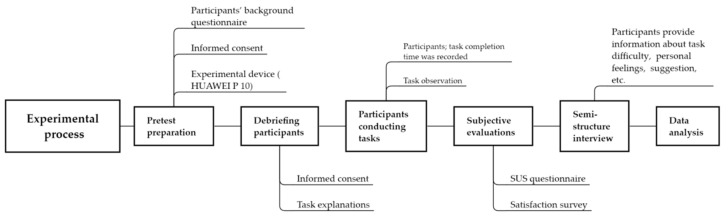
The experimental process of this study.

According to the independent variable, the participants were divided into three groups. Each participant was required to use one of the three operation modes, that is, the Litchi, Himalayas, and Archimedes types. The collection of the research data adopted both quantitative and qualitative tools. More specifically, when the experiment was conducted, the participant was asked to sit in front of a table. There were informed consent, personal background information questionnaire, and task descriptions on the table. After the participant filled in the information and understand the task features, s/he was given a mobile phone to conduct the assigned task in sequence (see Table 1) and each of the task completion time was recorded for further analysis. Then s/he was asked to fill out the SUS and subjective evaluation questionnaires. In the end, a semi-structure interview was also conducted to help collect the information pertinent to his/her task difficulty, personal feelings, etc. Detailed experimental process is provided as Figure 3.

More specifically, during the experiment, the participants were randomly assigned to three groups to test one of the three FM new media user interfaces. The participants were informed that they should perform five tasks in sequence as quickly and accurately as possible. After completing all the tasks, they were required to fill out the System Usability Scale (SUS) questionnaire (see Table 2). Each item of the SUS questionnaire was scored using a five-point Likert scale (from 1 “strongly disagree” to 5 “strongly agree”). After that, participants were also asked to complete a subjective evaluation questionnaire regarding FM new media app. The questionnaire was designed based on a 7-point Likert scale (from 1 for less satisfied to 7 for greatly satisfied). Finally, we conducted semi-structured interviews with participants on related issues. The interview mainly focused on overall user experience about the interface design and design suggestions. The total experiment time was less than 45 min.

## 4. Results and Analysis

A one-way analysis of variance (ANOVA) was performed to help analyze the collected data. The results generated from the one-way ANOVA of each task pertinent to participants’ completion time are illustrated in Table 3.

### 4.1. Task Analysis

As shown in Table 3, the first task required the participants to “Query view list”. The effect of the operation mode showed a significant difference (F (2, 27) = 6.375, *p* = 0.005 < 0.05). The subsequent post hoc comparison showed that the Himalayas type (M = 54.20, SD = 26.27) and the Litchi type (M = 103.32, SD = 28.04) had a significant difference (*p* = 0.012 < 0.05). The Himalayas type and the Archimedes type (M = 116.07, SD = 59.56) also showed a significant difference (*p* = 0.001 < 0.05). The Litchi type and the Archimedes type showed no significant effect (*p* = 0.492 > 0.05). The results indicated that the Himalayas type operation time was the shortest, and the Archimedes and Litchi types were the longest. According to results of the observations and interviews, the reason could be that participants using the Himalayas type interface can see the position of the ranking check so that they can quickly complete the task. However, the other two types require more than two additional steps to perform the task, and the task-related information was not obvious, which made the implementation of the tasks not intuitive. This is also because the lack of compelling visual cues can lead to reduced search and click performance [21].

The second task required the participants to “search for audiobooks”. The results showed that the effect of the operation mode showed no significant difference (F (2, 27) = 1.043, *p* = 0.366 > 0.05).

The third task required the participants to “select download content”. The effect of the operation mode showed no significant difference (F (2, 27) = 0.943, *p* = 0.402 > 0.05).

The fourth task required the participants to “delete the downloaded content”. The effect of the operation mode showed a significant difference (F (2, 27) = 5.242, *p* = 0.012 < 0.05). The subsequent post hoc comparison showed that the Himalayas type (M = 16.23, SD = 7.80) and the Litchi type (M = 35.80, SD = 23.33) had no significant difference (*p* = 0.053 > 0.05). The Himalayas type and the Archimedes type (M = 47.24, SD = 28.32) showed a significant difference (*p* = 0.003 < 0.05). The Litchi type and the Archimedes type also showed no significant difference (*p* = 0.248 > 0.05). The results indicated that the Himalayas type operation time was the shortest, and the Archimedes type was the longest. According to observations and interviews, the reason could be that the download indicator on the Himalayas type interface is in the upper right corner of the homepage. The operation tasks tend to be intuitive, making it convenient to delete information. The download button of the Archimedes type may not be obvious to the user. When searching for content, a user will need to perform multiple clicking steps. Only well-designed icons can convey system intent [17].

The fifth task required the participants to “share songs to WeChat moments”. The effect of the operation mode showed no significant difference (F (2, 27) = 1.173, *p* = 0.325 > 0.05).

### 4.2. System Usability Scale (SUS)

The participants were asked to fill out the SUS questionnaire after they had completed the tasks. The results generated from the one-way ANOVA are shown in Table 4.

From Table 4, it can be seen that there existed a significant difference in the effect of the operation mode (F (2, 27) = 7.067, *p* = 0.003 < 0.05). The subsequent post hoc comparison showed that the Himalayas type (M = 69.60, SD = 14.31) and the Litchi type (M = 45.50, SD = 18.55) had a significant difference (*p* = 0.012 < 0.05). The Himalayas type and the Archimedes type (M = 37.50, SD = 25.52) showed a significant difference (*p* = 0.001 < 0.05). The Litchi type and the Archimedes type also showed no significant difference (*p* = 0.376 > 0.05). That is, the Himalayas type was better than the Litchi and Archimedes types. According to the system usability scale (SUS), the evaluation greater than 68 means that it met the users’ needs and approval, while the operation mode below 68 indicated that the participant was not satisfied. The participants were satisfied with the Himalayas type (i.e., 69.60 > 68). However, the Litchi and Archimedes types both scored below 68, which showed that they were not satisfied with those two types.

The reason for the participants’ dissatisfaction may be that the ease-of-use of the system affects users’ perception of operation, and it can help them complete the operation process quickly and smoothly. Well-designed products follow the principles of interaction design and are user-centric. The Himalayas type operating system is in line with the participants’ visual perception of FM new media. Its interface visual clues or displayed information are hierarchical and easy to understand, which can effectively guide the participants to recognize the highlight of design characteristics regarding cognitive affordance. The object affordance of the operation interface mode is helpful for implementing the task. The function icons and text design consistently affect the effectiveness of the system function, while its functional affordance can help participants complete the task effectively. The design features of the size of icons, text, and buttons promote participants’ sense of affordance, which is beneficial to user perception and search tasks. Therefore, the Himalaya type has the design characteristics of the four factors of affordance, and participants can perceive the ease-of-use of the interface operating system and facilitate smooth interaction [14].

### 4.3. Subjective Evaluations

By using a 7-point Likert scale (i.e., from 1 for less satisfied to 7 for greatly satisfied), the results of the participants’ subjective evaluations after completing the operational tasks are presented as follows.

Table 5 illustrates the one-way ANOVA results of “interface aesthetics”, “search convenience”, “download intuitiveness”, “ease of deleting content”, “information reading fluency”, “ease of use”, “ease of query”, “functional convenience” and “overall acceptance” of the interface based on the participants’ subjective evaluation.

For “interface aesthetics”, the effect of the operation mode showed no significant difference (F (2, 27) = 1.024, *p* = 0.373 > 0.05). By comparing the averages, they show that participants’ total score for the Litchi (M = 3.50, SD = 1.18) and Archimedes (M = 3.40, SD = 1.26) types are less than 4, and the Himalayas (M = 4.10, SD = 1.10) type is more than 4. Therefore, participants’ subjective evaluation results pertinent to interface aesthetics tended to be neutral.

For “search convenience”, the effect of the operation mode showed no significant difference (F (2, 27) = 0.515, *p* = 0.603 > 0.05). By comparing the averages, they show that participants’ total score for the Archimedes (M = 3.80, SD = 2.10) type is less than 4, and the Litchi (M = 4.00, SD = 1.33) and Himalayas (M = 4.50, SD = 1.18) types are equal to or more than 4. Therefore, participants’ subjective evaluation results pertinent to search convenience also tended to be neutral.

For “download intuitiveness”, the effect of the operation mode showed no significant difference (F (2, 27) = 2.377, *p* = 0.112 > 0.05). By comparing the averages, they showed that participants’ total score for the Litchi (M = 3.60, SD = 1.17) type is less than 4, and the Himalayas (M = 4.80, SD = 1.03) and Archimedes (M = 4.40, SD = 1.51) types were more than 4. Therefore, participants’ subjective evaluation results pertinent to download intuitiveness also tended to be neutral.

For “ease of deleting content”, the effect of the operation mode showed no significant difference (F (2, 27) = 1.518, *p* = 0.237 > 0.05). By comparing the averages, they showed that participants’ total score for the Litchi (M = 4.30, SD = 1.77), Himalayas (M = 5.30, SD = 1.16) and Archimedes (M = 4.20, SD = 1.69) types were all more than 4. According to the evaluation criteria of the 7-point Likert scale, the participants were all satisfied with the three operation modes.

For “information reading fluency”, the effect of the operation mode showed a significant difference (F (2, 27) = 4.134, *p* = 0.027 < 0.05). The subsequent post hoc comparison showed that the Himalayas type (M = 5.20, SD = 1.32) and the Litchi type (M = 3.10, SD = 1.73) had a significant difference (*p* = 0.008 < 0.05). The Himalayas type and the Archimedes type (M = 4.00, SD = 1.83) showed no significant difference (*p* = 0.113 > 0.05). The Litchi type and the Archimedes type also showed no significant difference (*p* = 0.230 > 0.05). The results indicated that the Himalayas type operation time had the highest satisfaction in terms of information reading fluency, and the Litchi type had the lowest satisfaction. The reason may be that the Litchi type lacks an overall information column, and the difference between the information columns of each module is not clear, which also causes reading difficulties. Due to the limitation of the screen size of the mobile phone, the interface information might be too small for users to easily pay attention to the displayed content.

For “ease of use”, the effect of the operation mode showed a significant difference (F (2, 27) = 4.134, *p* = 0.027 < 0.05). The subsequent post hoc comparison showed that the Himalayas type ((M = 4.40, SD = 1.17) and the Litchi type (M = 2.90, SD = 1.66) had a significant difference (*p* = 0.029 < 0.05). The Himalayas type and the Archimedes type (M = 2.80, SD = 1.48) also showed a significant difference (*p* = 0.020 < 0.05). The Litchi type and the Archimedes type showed no significant difference (*p* = 0.879 > 0.05). The results indicated that the Himalayas type had the highest satisfaction in terms of ease of use. The Litchi and Archimedes types had the lowest satisfaction. The reason may be that the overall arrangement of the Himalayas type interface information is more systematic and well organized. When the content is rich and rationally arranged, it might enhance participants’ awareness of interface information and highlight the design characteristics regarding cognitive affordance.

For “ease of query”, the effect of the operation mode showed a significant difference (F (2, 27) = 4.134, *p* = 0.027 < 0.05). The post hoc comparison showed that the Himalayas type (M = 4.80, SD = 1.32) and the Litchi type (M = 2.80, SD = 1.44) had a significant difference (*p* = 0.004 < 0.05). The Himalayas type and the Archimedes type (M = 3.50, SD = 1.58) showed no significant difference (*p* = 0.053 > 0.05). The Litchi type and the Archimedes type also showed no significant difference (*p* = 0.285 > 0.05). The results indicated that the Himalayas type operation time had the highest satisfaction in terms of ease of query, and the Litchi type had the lowest satisfaction.

For “functional convenience”, the effect of the operation mode showed no significant difference (F (2, 27) = 1.664, *p* = 0.208 > 0.05). By comparing the averages, they showed that participants’ total score for the Litchi (M = 4.20, SD = 1.99), Himalayas (M = 5.40, SD = 1.78) and Archimedes (M = 4.10, SD = 1.52) types are all more than 4. According to the evaluation criteria of the 7-point Likert scale, the participants were all satisfied with the three operation modes.

For “overall acceptance of the interface”, the effect of the operation mode showed no significant difference (F (2, 27) = 2.291, *p* = 0.121 > 0.05). By comparing the averages, they show that participants’ total score for the Archimedes (M = 3.20, SD = 1.14) type was less than 4, and the Litchi (M = 4.00, SD = 1.15) and Himalayas (M = 4.40, SD = 1.51) types were equal to or more than 4. Therefore, participants’ subjective evaluation results pertinent to download intuitiveness also tended to be neutral.

## 5. Discussion

Previous research has shown that the perceived affordance of new media interaction design is multi-dimensional [9]. Users have the ability to implement intuitive interaction through the user interface design [7]. The four types of affordance concept expansions include cognition, function, sensory, and object affordance [14], which contribute to the interactive experience of users with new media. Based on the experimental results, the analyzed results are addressed as follows.

### 5.1. Participants’ Task Performance

In terms of task performance, the three operating interfaces of FM new media have significant differences. The Himalayas task performance takes the shortest time, and participants can quickly complete the assigned tasks. This means that the user interface is recognizable and easy to operate because of higher affordance. We can learn from the analysis of the four types of affordance.

Cognitive affordance emphasizes a simple and easy-to-operate interface design. Interface graphics and text follow the principle of consistency, and the use of familiar icons and text can stimulate the cognitive level of the brain [24]. Affordance clues can affect search and click on web buttons to call-action [21]. They can fully mobilize the user’s subjective initiative and participate in the interaction. For example, on the function to delete the downloaded content and the sliding prompt, the interface configuration of the primary download information and the secondary download information should be simple, intuitive, and logical. When the hidden functions of different levels slide the extended information in the horizontal and vertical directions, it should also provide easy-to-recognize guidance prompts, which can effectively and quickly help participants delete the content function.

Object affordance refers to the possibility of helping users take action. Interface design is an active device for the new media of smart touch screens. When users interact with the new media interface, although there is no explicit object affordance, it is the stealth meaning of the touch screen interface. This includes icons, icon size, shadow, color, and so on. and can provide stealth affordance. One possible explanation is that the size of the touch screen also affects the interaction [25], and the corresponding interface design elements are displayed through invisible energy, and are designed appropriately to trigger users to take action. The close connection between smartphone screen size and interface elements is directly related to usability issues. For example, a larger button can more easily trigger action than a smaller one. Moreover, when the finger is too fast, the size of the icon on the touch screen may affect the degree of input failure [26]. At the same time, it may also affect user safety, task, and satisfaction. The precise input method corresponds to the content of the link interface, system settings, and interactive group status [25].

The intuitiveness and clarity of functional affordance can help users quickly complete the FM interface operation tasks. In terms of usability function configuration, we learned through “Checking the music rankings” that the display functions effect of the Litchi and Archimedes types are lower than that of the Himalayas type. This may be related to the inconsistency of the interface information, resulting in the information content being not sufficiently intuitive, and needing a specific search function to see the relevant information. It not only affects the user’s operability but also affects visibility and makes reading more difficult. On the function menu, the graphics and text should be designed with more consistent and clear considerations. On the Litchi and Archimedes user interfaces, the meaning of the words can easily confuse the audience, so that it is impossible to interpret the relationship between the sound and the program.

### 5.2. Participants’ Subjective Evaluation

This research study further examines the impact of the three operation interfaces on the subjective evaluation of users. The overall results showed that there are significant differences between the three operating interfaces, with the participants unanimously rating Himalayas better than Litchi and Archimedes. One possible explanation is that the Himalayas interface is easier to use, and the integration of information, functions, and icons is more systematic and well organized. It not only considers the usability of the design but also takes into account the social nature of the emotional needs of new media. It also follows the principles of interaction design, pays attention to the overall effect of the interface, avoids multiple clicks on the functional structure, and provides clear and intuitive semantic transmission. As an FM listening platform, the functions of “interface query view”, “music ranking list”, and “click to play order” are all frequent options for users. The overall information structure of the interface should conform to the public perception and interaction logic. For instance, the searching for personal data and downloading and storing information should be in the most conspicuous location, and the interface texts should be clear. When a user searches for Archimedes personal information, the semantic expression might not be sufficiently intuitive, and the display position tended to be invisible. Given the above analysis, designers should consider the usability of users from multiple perspectives, and strengthen the visual affordance of the interface according to user needs to help enhance the user’s interactive experience from unfamiliar to familiar status.

## 6. Conclusions

The focus of this study was on applying the concept of affordance and its factors to improve the usability of the FM new media communication platform. The study analyzed the current situation of new media and the impact of the application of affordance factors regarding the design of a new media interface. The result shows that the concept of affordance has a significant impact on users’ task performance of the FM new media operation mode, and affects the subjective evaluation of users. We summarize the interface design of the FM smart app platform and provide several suggestions as follows.

There are significant differences between the three user interfaces, where the Himalayas type is better than the Litchi and Archimedes types. That is, the participants’ task performance takes the shortest time. The subjective evaluation results show that the three user interfaces also have significant differences. Participants are most satisfied with the Himalayas type as it meets the needs of the users. The overall results show that the visual affordance directly affects the user’s perception, and effectively improves the user’s task performance and subjective evaluations.

To improve the effective development of FM new media products, we put forward the following points based on the summarized results of the FM platform interface design. The FM new media interface is the communication window of human-computer interaction. Designers should give full consideration to user needs, simplify interface operation complexity, provide clear and easy-to-recognize information content, and mobilize design functions, visual senses, and object affordance applications [6]. It is important to take into account both the usability and social benefits of the FM new media platform.

In addition, the user’s intuition with the smart touch screen interface during the use of FM new media is not only affected by the size of the active device’s touch screen, but also by its interface design. Therefore, designers need to consider direct or indirect object affordance to improve invisible affordance design. When traditional media are switching to new media, users turn from passive to active behavior, and perform tasks through visual cues or information. Enhancing the cognitive affordance of the interface design can help guide users to perform tasks. It is also important to clarify the interface information and help users complete tasks through functional icons. Designers should follow the principle of consistency in graphic design, make full use of the information that users are familiar with, and use the affordance design concept to improve user experiences. It is recommended that future research can be combined with the current high-profile VR/AR to help better understand user needs and enhance interactivity. At the same time, incorporating studies pertinent to the facial expression of social robots (e.g., Song & Luximon [27]) can also help design better innovative media platform to promote social interactions.

## Figures and Tables

**Table 1:** The experimental tasks of FM new media apps.

Task Type	Task Description
Task1: Check the music chart	Please to query the FM interface music rankings, click the first place, then click the fifth place.
Task2: Search for audiobooks	Please search for audiobooks “passing by your world”, click play, click pause.
Task3: Select download content	Please select “Passing by your world” to download the second content.
Task4: Delete the downloaded content	Please go back to the homepage, find the downloaded page, and delete the “Passing by Your World” audiobook.
Task5: share songs to WeChat moments	Please find the music from the category and share the third music.

**Table 2:** The Likert scale items of system usability scale (SUS) questionnaire.

No.	Questions
1	I think that I would like to use this APP system frequently.
2	I found the APP system unnecessarily complex.
3	I thought the APP system was easy to use.
4	I think that I would need the support of a technical person to be able to use this APP system.
5	I found the various functions in this APP system were well integrated.
6	I thought there was too much inconsistency in this APP system.
7	I would imagine that most people would learn to use this APP system very quickly.
8	I found the APP system very cumbersome to use.
9	I felt very confident using the APP system.
10	I needed to learn a lot of things before I could get going with this APP system.

**Table 3:** The one-way ANOVA result of each task regarding participants’ task completion time.

Source	SS	DF	MS	F	*p*	Post Hoc (LSD)
Task 1	213,487.783	2	10,674.391	6.375	0.005 *	Himalayas < Litchi = Archimedes
Task 2	1834.263	2	917.302	1.043	0.366	
Task 3	319.348	2	159.674	0.943	0.402	
Task 4	4917.979	2	2458.990	5.242	0.012 *	Himalayas < Archimedes
Task 5	494.357	2	1.173	1.173	0.325	

* Significantly different at α = 0.05 level (* *p* < 0.05).

**Table 4:** The one-way ANOVA result of SUS questionnaire.

Source	SS	DF	MS	F	*p*	Post Hoc (LSD)
SUS	5584.000	2	2792.033	7.067	0.003 *	Himalayas < Litchi = Archimedes

* Significantly different at α = 0.05 level (* *p* < 0.05).

**Table 5:** The results of the one-way ANOVA regarding participants’ subjective evaluations.

Source	SS	DF	MS	F	*p*	Post Hoc (LSD)
Interface aesthetics	2.867	2	1.433	1.024	0.373	
Search convenience	2.600	2	1.300	0.515	0.603	
Download intuitiveness	7.467	2	3.733	2.377	0.112	
Ease of deleting content	7.400	2	3.700	1.518	0.237	
Information reading fluency	22.200	2	11.100	4.134	0.027 *	Himalayas < Litchi < Archimedes
Ease of use	16.067	2	8.033	3.812	0.035 *	Himalayas < Litchi < Archimedes
Ease of query	20.600	2	10.300	4.993	0.014 *	Himalayas < Litchi < Archimedes
Functional convenience	10.467	2	5.233	1.664	0.208	
Overall acceptance	7.467	2	3.733	2.291	0.121	

* Significantly different at α = 0.05 level (* *p* < 0.05).
